# International Comparison of Underlying Disease Among Recipients of Medical Assistance in Dying

**DOI:** 10.1001/jamainternmed.2024.6643

**Published:** 2024-12-09

**Authors:** Brandon Heidinger, Colleen Webber, Kenneth Chambaere, Eliana Close, Luc Deliens, Bregje Onwuteaka-Philipsen, Thaddeus Pope, Agnes van der Heide, Ben White, James Downar

**Affiliations:** 1 Ottawa Hospital Research Institute, Ottawa, Ontario, Canada; 2 End-of-Life Care Research Group, Ghent University, Ghent, Belgium; 3 Vrije Universiteit Brussel, Ghent University, Ghent, Belgium; 4 Australian Centre for Health Law Research, Faculty of Business and Law, Queensland University of Technology, Brisbane, Australia; 5 Department of Public and Occupational Health, Amsterdam UMC, location VU Medical Center, Amsterdam, the Netherlands; 6 Mitchell Hamline School of Law, Saint Paul, Minnesota; 7 Department of Public Health, Erasmus MC, University Medical Center Rotterdam, Rotterdam, the Netherlands; 8 Division of Palliative Care, Department of Medicine, University of Ottawa, Ottawa, Ontario, Canada

## Abstract

This cohort study compares the rates of medical assistance in dying across diseases to understand the relative effects of disease and jurisdiction.

In 2023, 282 million individuals lived in jurisdictions allowing medical assistance in dying (MAID). Eligibility criteria (eg, prognosis) and type of MAID (self-administered MAID or physician-assisted suicide [PAS] vs clinician-administered MAID or euthanasia) vary by jurisdiction, with MAID use ranging from 0.1% to 5.1% of all deaths.^1^ But regardless of jurisdiction, cancer and amyotrophic lateral sclerosis (ALS) consistently account for up to 80% of MAID cases^1,2^ despite accounting for fewer than 30% of all deaths.^3^ To better understand the relative effect of disease and jurisdiction on the use of MAID, we conducted an international comparison of the rates of MAID deaths across diseases.

## Methods

We collected publicly available data for 20 jurisdictions with legal MAID and compared MAID deaths as a proportion of all deaths for the most frequent underlying diseases (eMethods and eTables 1 to 4 in Supplement 1). We also compared MAID deaths for lung vs nonlung cancers. We included 5 jurisdictions in Australia, Belgium, Canada, Luxembourg, the Netherlands, New Zealand, and Switzerland and 9 jurisdictions in the US. We used negative binomial regression to model the count of the number of MAID deaths, with disease group, year, and jurisdiction included as fixed effects and the total number of deaths included as an offset term. This study was exempt from institutional review board approval because only publicly available, deidentified, aggregate data were used. All analyses were conducted using SAS version 9.4 (SAS Institute).

## Results

We collected data from 20 jurisdictions representing 184 695 MAID deaths and 12 933 459 total deaths between 1999 and 2023 (Table). A total of 10 of the included jurisdictions (50%) allowed for MAID by PAS only, 15 (75%) required an estimated life expectancy of less than 6 to 12 months, and 17 (85%) required a minimum age of 18 years. Cancer was the underlying disease for 122 759 MAID recipients (66.5%), followed by nervous system disease (14 941 [8.1%]), circulatory system disease (12 478 [6.8%]), and respiratory system disease (8968 [4.9%]). By condition, the share receiving MAID was highest for ALS (2967 of 17 631 deaths [16.8%] in all jurisdictions), followed by cancer (122 759 of 3 277 368 deaths [3.7%]). Compared with Belgium, the proportion of MAID deaths, controlling for underlying disease and year, was highest in the Netherlands (risk ratio [RR], 1.96; 95% CI, 1.74-2.21) and lowest in New Jersey (RR, 0.04; 95% CI, 0.03-0.06) (Figure, A). People dying of ALS were more likely to receive MAID (RR, 6.84; 95% CI, 5.63-8.32) than people with cancer, while people with nervous system disease excluding ALS (RR, 0.33; 95% CI, 0.27-0.41), respiratory system disease (RR, 0.25; 95% CI, 0.21-0.29), and circulatory system disease (RR, 0.09; 95% CI, 0.07-0.10) were less likely to receive MAID than people with cancer. People dying of lung and nonlung cancers were equally likely to receive MAID. Jurisdictions that allowed euthanasia had higher rates of MAID than those that allowed only PAS (RR, 2.70; 95% CI, 2.37-3.08), and those with no prognostic requirement had higher rates than those with a prognostic requirement (RR, 2.14; 95% CI, 1.87-2.44) (Figure, B). The overall incidence of MAID increased over time (per 1 calendar year: RR, 1.11; 95% CI, 1.10-1.11).

**Table.** Medical Assistance in Dying (MAID) and Overall Mortality by Jurisdiction Across All Years and in the Most Recent Reporting Period

Jurisdiction	Year	All years with data			Most recent reporting period		
		MAID deaths, No.^a^	All deaths, No.^a^	MAID incidence, %	MAID deaths, No.^a^	All deaths, No.^a^	MAID incidence, %
Australia							
Queensland	2023	245	17 759	1.4	245	17 759	1.4
South Australia	2023	39	6012	0.6	39	6012	0.6
Tasmania	2022-2023	25	2564	1.0	25	2564	1.0
Victoria	2019-2023	912	174 139	0.5	306	47 978	0.6
Western Australia	2021-2023	446	33 190	1.3	255	17 299	1.5
Belgium	2003-2022	30 194	2 151 699	1.4	2966	112 291	2.6
Canada	2017-2022	44 958	2 033 601	2.2	13 241	304 970	4.3
Luxembourg	2015-2022	170	55 935	0.3	34	4338	0.8
The Netherlands	2004-2022	88 046	2 764 985	3.2	8720	169 938	5.1
New Zealand	2022-2023	328	32 783	1.0	328	32 783	1.0
Switzerland	1999-2018	8738	1 261 923	0.7	4820	330 567	1.5
US							
California	2016-2022	3349	2 058 520	0.1	853	334 817	0.3
Colorado	2017-2022	1090	259 179	0.4	316	48 284	0.7
Hawaii	2019-2021	111	36 487	0.3	49	12 895	0.4
Maine	2020-2022	146	50 297	0.3	54	17 270	0.3
New Jersey	2019-2022	186	295 419	0.1	91	84 163	0.1
Oregon	1999-2022	2454	845 682	0.3	278	44 981	0.6
Vermont	2013-2021	116	49 753	0.2	29	12 546	0.2
Washington	2009-2022	3127	781 566	0.4	433	68 673	0.6
Washington, DC	2018-2021	15	21 966	0.1	6	5833	0.1
All jurisdictions	1999-2023	184 695	12 933 459	1.4	33 088	1 675 961	2.0

^a^ MAID deaths and all deaths included all reported deaths, regardless of the underlying disease.

**Figure.**
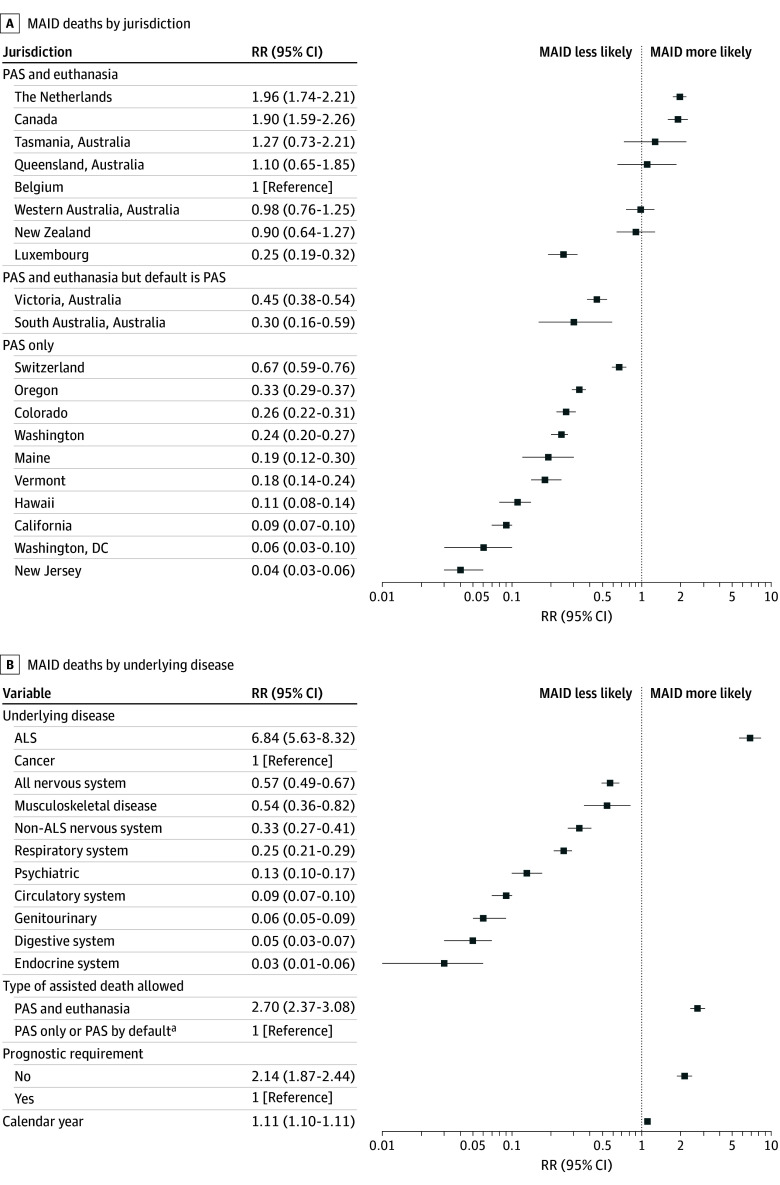
Relative Differences in Medical Assistance in Dying (MAID) Deaths by Jurisdiction and by Underlying Disease A, Relative difference in the proportion of MAID deaths by jurisdiction, controlling for disease and year. B, Relative difference in the proportion of MAID deaths by underlying disease, eligibility criteria, and year, excluding jurisdiction from the model. ALS indicates amyotrophic lateral sclerosis; PAS, physician-assisted suicide; RR, risk ratio. ^a^In Victoria and South Australia, legislation includes procedural steps making euthanasia much more challenging to access than PAS, so that more than 85% of MAID recipients used PAS. In contrast, in Western Australia, New South Wales, and Queensland, less than 35% use PAS.

## Discussion

While absolute MAID rates and eligibility criteria^1^ vary by jurisdiction, the relative proportion of MAID rates by disease was remarkably similar across jurisdictions. The difference in MAID rates across diseases was substantial—far greater than the difference accounted for by eligibility criteria or type of MAID permitted and greater than sociodemographic factors previously reported.^2,4,5^ Differences in MAID rate by disease also remained the same whether or not jurisdiction was included as a fixed effect in the model. This observation is consistent with the idea that MAID is driven heavily by illness-related factors common to people with those illnesses and inconsistent with the idea that MAID is driven substantially by factors that are external to the individual and that vary by jurisdiction, such as eligibility criteria, culture, social assistance, or palliative care service availability.^1,6^ This study was limited by its retrospective observational design, as well as inconsistencies in how MAID data are recorded and reported among various jurisdictions. The data are also mostly from European and Western jurisdictions with majority White populations; the international similarities may not extend to different populations. Temporal trends are similarly limited, as many jurisdictions have only recently legalized MAID.

## Notes

Supplement 1.
eMethods.
**eTable 1.** Jurisdictional MAID reports **eTable 2.** Jurisdictional mortality data **eTable 3.** Matched narrative descriptions of the most and least common underlying diseases for mortality ICD-10 chapters and subchapters by jurisdiction **eTable 4.** MAID eligibility laws and regulations by jurisdiction

Supplement 2. Data Sharing Statement
