# Supplementary material for: International Comparison of Underlying Disease Among Recipients of Medical Assistance in Dying
Source: JAMA Intern Med. 2024 Dec 9;185(2):235–7. doi: 10.1001/jamainternmed.2024.6643 (PMC11791694; doi:10.1001/jamainternmed.2024.6643)
Supplement: Supplement 1. — eMethods. eTable 1. Jurisdictional MAID reports eTable 2. Jurisdictional mortality data eTable 3. Matched narrative descriptions of the most and least common underlying diseases for mortality ICD-10 chapters and subchapters by jurisdiction eTable 4. MAID eligibility laws and regulations by jurisdiction [file jamainternmed-e246643-s001.pdf]

Supplemental Online Content

Heidinger B, Webber C, Chambaere K, et al. International comparison of underlying disease among recipients of medical assistance in dying. *JAMA Intern Med*. Published online December 9, 2024. doi:10.1001/jamainternmed.2024.6643

eMethods.

eTable 1. Jurisdictional MAID reports

eTable 2. Jurisdictional mortality data

eTable 3. Matched narrative descriptions of the most and least common underlying diseases for mortality ICD-10 chapters and subchapters by jurisdiction

eTable 4. MAID eligibility laws and regulations by jurisdiction

This supplementary material has been provided by the authors to give readers additional information about their work.

© 2024 Heidinger B et al. *JAMA Internal Medicine*.

eMethods.

Setting:

The following jurisdictions were included in our analysis: Australia – Queensland, Australia – South Australia, Australia – Tasmania, Australia – Victoria, Australia – Western Australia, Belgium, Canada, Luxembourg, the Netherlands, New Zealand, Switzerland, US – California, US – Colorado, US – DC, US – Hawaii, US – Maine, US – New Jersey, US – Oregon, US – Vermont, US – Washington.

Some jurisdictions have national laws that allow MAID, whereas others, such as the United States and Australia, have differing MAID laws by state. Spain and Colombia have legal MAID, but were not included in this study as they did not have publicly available MAID data at the time of our analysis. **eTable 1** included sources for MAID reports by jurisdiction.

As Switzerland does not publish national MAID reports, we used data from a Swiss study that analyzed cause of death statistics for all deaths among Swiss permanent residents that occurred between 1999 to 2018 in Switzerland. To capture MAID deaths with ALS as the underlying disease in the Netherlands, we used data from a 2023 study covering all ALS MAID deaths between 2012 and 2020.

MAID decedents:

For each jurisdiction, we recorded the total number of MAID deaths by year for all years with available data. We extracted this data from published MAID reports. We separately recorded the number of MAID deaths by year with the most frequently reported underlying diseases, including cancer, nervous system disease, circulatory disease, respiratory disease, endocrine disease, psychiatric conditions, digestive system disease, musculoskeletal disease, and genitourinary disease. We also recorded MAID deaths from amyotrophic lateral sclerosis (ALS), or MAID deaths from motor neuron disease if the number of ALS-specific deaths was not available. Finally, we recorded the number of MAID decedents with lung cancer, which is the most frequently reported cancer site among MAID recipients. Data availability varied by

© 2024 Heidinger B et al. *JAMA Internal Medicine*.

jurisdiction and reporting year, with many earlier reports only including broad categories of underlying diagnoses (e.g., cancer vs. nervous system disease vs. other).

In most cases, MAID data included the underlying disease of those who received MAID. However, some jurisdictions only reported the underlying diseases of those who applied for MAID, regardless of whether they were found to be eligible (i.e., Queensland, Victoria, and New Zealand); those who were deemed eligible after an initial assessment but not necessarily further assessments, (i.e., Western Australia), or those who were deemed eligible for MAID but did not necessarily receive MAID (i.e., Tasmania and Colorado). MAID data including underlying disease from the aforementioned jurisdictions was combined for all analyses.

All decedents:

From each jurisdiction with available MAID data, we recorded yearly mortality data extracted from public death registrars and reports. When available, we recorded the total number of deaths, as well as deaths corresponding to the following International Classification of Diseases 10th Revision (ICD-10) chapters: neoplasms (C00-D48), endocrine, nutritional and metabolic diseases (E00-E90), mental and behavioural disorders (F00-F99), diseases of the nervous system (G00-G99), diseases of the circulatory system (I00-I99), diseases of the respiratory system (J00-J99), diseases of the digestive system (K00-K93) diseases of the musculoskeletal system and connective tissue (M00-M99), and diseases of the genitourinary system (N00-N99). We also recorded Amyotrophic Lateral Sclerosis (ALS) (G12.21), or motor neuron disease (G12.2) if data for G12.21 was not available. Finally, we recorded lung cancer deaths, which corresponded to the ICD-10 Code “malignant neoplasm of bronchus and lung” (C34).

We compared data on the underlying disease of MAID recipients with data on population mortality cause of death. Most MAID reports used narrative diagnostic categories, such as “neurological conditions”, “cancer”, and “cardiovascular conditions,” whereas population mortality data are

© 2024 Heidinger B et al. *JAMA Internal Medicine*.

categorized using ICD-10 codes. We compared the narrative definitions in MAID deaths to their relevant ICD-10 chapters or subchapters for population mortality as appropriate (e.g., MAID deaths reported with an underlying disease of "neurological conditions" were compared with total deaths due to diseases of the nervous system, ICD-10 G00-G99). For example, Luxembourg's MAID report included a MAID underlying disease category of "neurovascular disease", which we included in the "diseases of the circulatory system" subchapter, whereas deaths with an underlying diagnosis of "neurodegenerative disease" were included with "diseases of the nervous system".

We recorded and analyzed MAID and mortality data by calendar year. When MAID reporting periods cut across calendar years (e.g., Western Australia- July 1st, 2021, to June 30th, 2022), we compared this data to mortality data from the closest available calendar year (i.e., January 1st, 2021 to December 31st, 2021). For example, we compared Canada 2022 MAID data with 2021 mortality data, as mortality data for 2022 was not released at the time of analysis.

When MAID reporting periods included partial years (i.e., when a jurisdiction initiated a MAID program in the middle of the year), such as New Jersey from August 1st, 2019 to December 31st, 2019, this data was duration-matched with available mortality data as closely as possible. For example, to duration-match data for New Jersey's first reporting period, as it only included 152 days, we used a fraction of 152/365 of 2019 deaths.

Finally, for jurisdictions that release MAID reports spanning multiple years, we used the previously described methods to match data by duration and time. For example, Victoria published a MAID report from June 19th, 2019, to June 30th, 2023, spanning four years and twelve days. To calculate the number of duration-matched deaths, we added together 2019 deaths, 2020 deaths, 2021 deaths, 2022 deaths, and a fraction of 12/365 of 2019 deaths. MAID data was excluded from our analyses of relative risk over time if it came from reports spanning multiple years.

© 2024 Heidinger B et al. *JAMA Internal Medicine*.

Statistical Analysis

We used descriptive statistics to characterize overall MAID incidence by jurisdiction and underlying disease. To compare MAID proportion by underlying disease, we calculated the proportion of each disease group that received MAID, and then calculated the relative risk of receiving MAID for each disease by dividing the MAID proportion for each disease by the MAID proportion for all cancer diagnoses (the reference value) using a multilevel negative binomial regression model. Jurisdictions were modelled as a random effect due to the clustering of observations within jurisdictions, and independent variables included underlying disease and year (recognizing that some jurisdictions had many more years of data than others). We then created forest plots showing the relative risk of receiving MAID for each diagnosis compared with all cancer diagnoses, as well as showing how these relative risks compare across jurisdictions. We also used regression models to calculate how MAID by underlying disease changes over time.

We also included a model analyzing the effect of the type of MAID available (PAS only vs. PAS and Euthanasia) and the presence or absence of a prognostic criterion. We had to remove jurisdiction from this model, as there was considerable overlap between these two factors among jurisdictions.

Ethics

This study did not require Research Ethics Board approval as per Ottawa Health Science Network – Research Ethics Board policy SOP 102.003.

© 2024 Heidinger B et al. *JAMA Internal Medicine*.

eTable 1. Jurisdictional MAID reports

Jurisdiction	Title of Most Recent MAID Report	Reporting Period of Most Recent MAID Report	Source
Australia – Queensland	Voluntary Assisted Dying Review Board Annual Report 2022-2023	January 1, 2023 – June 30, 2023	https://www.health.qld.gov.au/__data/assets/pdf_file/0024/1261185/vad-annual-report-2022-23.pdf
Australia – South Australia	Voluntary Assisted Dying Review Board Annual Report 2022-2023	January 31, 2023 – June 30, 2023	https://www.sahealth.sa.gov.au/wps/wcm/connect/c502e159-2307-4b27-9968-a06c41d219ad/23116.1+Voluntary+Assisted+Dying+Annual+Report_V9_WEB3.pdf?MOD=AJPERES&CACHEID=ROOTWORKSPACE-c502e159-2307-4b27-9968-a06c41d219ad-oLmvJkb
Australia – Tasmania	Voluntary Assisted Dying in Tasmania Voluntary Assisted Dying Commission Annual Report 2022-2023	October 23, 2022 – June 30, 2023	https://www.health.tas.gov.au/sites/default/files/2023-10/voluntary_assisted_dying_commission_annual_report_2022-23.pdf
Australia – Victoria	Voluntary Assisted Dying Review Board Annual Report July 2022 to June 2023	July 1, 2022 – June 30, 2023	https://www.safercare.vic.gov.au/sites/default/files/2023-08/VADRB%20Annual%20Report%202022-23.pdf
Australia – Western Australia	Voluntary Assisted Dying Board Western Australia Annual Report 2022-23	July 1, 2022 – June 30, 2023	https://www.health.wa.gov.au/~/media/Corp/Documents/Health-for/Voluntary-assisted-dying/VAD-Board-Annual-Report-2022-23.pdf
Belgium	Communiqué de presse de la Commission fédérale de Contrôle et d'Évaluation de l'Euthanasie – CFCEE EUTHANASIE – Chiffres de l'année 2022	January 1, 2022 – December 31, 2022	https://organesdeconcertation.sante.belgique.be/fr/documents/euthanasie-chiffres-de-lannee-2022
Canada	Fourth annual report on Medical Assistance in Dying in Canada 2022	January 1, 2022 – December 31, 2022	https://www.canada.ca/en/health-canada/services/publications/health-system-services/annual-report-medical-assistance-dying-2022.html
Luxembourg	Septième rapport de la loi du 16 mars 2009 sur l'euthanasie et l'assistance au suicide (années 2021 et 2022)	January 1, 2021 – December 31, 2022	https://sante.public.lu/fr/publications/r/rapport-euthanasie-2021-2022.html
The Netherlands	Regional Euthanasia Review Committees Annual Report 2022	January 1, 2022 – December 31, 2022	https://english.euthanasiecommissie.nl/the-committees/documents/publications/annual-reports/2002/annual-reports/annual-reports

© 2024 Heidinger B et al. *JAMA Internal Medicine*.

The Netherlands	Frequency of euthanasia, factors associated with end-of-life practices, and quality of end-of-life care in patients with amyotrophic lateral sclerosis in the Netherlands: a population-based cohort study(20)	January 1, 2012 – December 31, 2020	Eenennaam RM van, Kruithof W, Beelen A, Bakker LA, Eijk RPA van, Maessen M, et al. Frequency of euthanasia, factors associated with end-of-life practices, and quality of end-of-life care in patients with amyotrophic lateral sclerosis in the Netherlands: a population-based cohort study. The Lancet Neurology. 2023 Jul 1;22(7):591–601.
New Zealand	Assisted Dying Service – Ngā Ratonga Mate Whakaahuru Registrar (assisted dying) Annual Report to the Minister of Health – June 2023	April 1, 2022 – March 31, 2023	https://www.health.govt.nz/system/files/documents/publications/registrar-assisted-dying-annual-report-2023-july23.pdf
Switzerland	Long-term experience on assisted suicide in Switzerland: dementia, mental disorders, age-related polymorbidity and the slippery slope argument(21)	January 1, 1999 – December 31, 2018	Güth U, Junker C, McMillan S, Elfgen C, Schneeberger AR. Long-term experience on assisted suicide in Switzerland: dementia, mental disorders, age-related polymorbidity and the slippery slope argument. Public Health. 2023 Oct 1;223:249–56.
US – California	California End of Life Option Act 2022 Data Report	January 1, 2022 – December 31, 2022	https://www.cdph.ca.gov/Programs/CHSI/CDPH%20Document%20Library/CDPH_End_of_Life%20_Option_Act_Report_2022_FINAL.pdf
US – Colorado	Colorado End-of-Life Options Act, 2022 Data Summary, with 2017-2022 trends and totals	January 1, 2017 – December 31, 2022	https://cdphe.colorado.gov/center-for-health-and-environmental-data/registries-and-vital-statistics/medical-aid-in-dying#Annual
US – DC	District of Columbia Death with Dignity Act 2022 Data Summary	February 19, 2021 – February 18, 2022	https://dchealth.dc.gov/sites/default/files/dc/sites/doh/publication/attachments/2022%20Death%20with%20Dignity%20Annual%20Report.pdf
US – Hawaii	Hawaii Department of Health (DOH) 2021 Our Care Our Choice Annual Report (DOH)	January 1, 2021 – December 31, 2021	https://health.hawaii.gov/opppd/files/2022/07/corrected-MAID-2021-Annual-Report.pdf
US – Maine	Report to The Maine Legislature Patient-Directed Care 2022 Annual Report	January 1, 2022 – December 31, 2022	https://www.mainedeathwithdignity.org/wp-content/uploads/2023/05/Patient-Directed-Care-Annual-Report-CY-2022.pdf
US – New Jersey	New Jersey Medical Aid in Dying for the Terminally Ill Act 2022 Data Summary	January 1, 2022 – December 31, 2022	https://www.nj.gov/health/advancedirective/documents/maid/MAidAnnualReport2022.pdf
US – Oregon	Oregon Death with Dignity Act Data 2021 Data Summary	January 1, 2022 – December 31, 2022	https://www.oregon.gov/oha/PH/PROVIDERPARTNERRESOURCES/EVALUATIONRESEARCH/DEATHWITHDIGNITYACT/Documents/year24.pdf
US – Vermont	Report to the Vermont Legislature	July 1, 2019 – June 30, 2021	https://legislature.vermont.gov/assets/Legislative-Reports/2022-Patient-Choice-Legislative-Report.Final.pdf

© 2024 Heidinger B et al. *JAMA Internal Medicine*.

	Report Concerning Patient Choice at the End of Life		
US – Washington	Report to the Legislature<br>2022 Death with Dignity	January 1, 2022 – December 31, 2022	https://doh.wa.gov/sites/default/files/2023-10/422-109-DeathWithDignityAct2022.pdf

© 2024 Heidinger B et al. *JAMA Internal Medicine*.

eTable 2. Jurisdictional mortality data

Jurisdiction	Source
Australia – Queensland, South Australia, Tasmania, Victoria, Western Australia	Australian Bureau of Statistics 2022. Causes of Death, Australia. Accessed August 27, 2024. https://www.abs.gov.au/statistics/health/causes-death/causes-death-australia/latest-release
Belgium	Statbel. Causes de décès. Accessed August 27, 2024. https://statbel.fgov.be/fr/themes/population/mortalite-et-esperance-de-vie/causes-de-deces#panel-13
Canada	Statistics Canada. Table 13-10-0145-01 Deaths, by cause, Chapter VI: Diseases of the nervous system (G00 to G99). Accessed August 27, 2024. https://doi.org/10.25318/1310014501-eng
Luxembourg	Saleh S., Abad D., Weiss J. (2023). Statistiques des causes de décès au Luxembourg pour l'année 2022, Direction de la santé, Luxembourg. sante.lu/statistiques-causes-deces
The Netherlands	CBS StatLine. Deaths; underlying cause of death (shortlist), sex, age. Accessed August 27, 2024. https://opendata.cbs.nl/statline/#/CBS/en/dataset/7052eng/table?dl=AB92B
The Netherlands (ALS)	Van Eenennaam RM, Kruithof W, Beelen A, et al. Frequency of euthanasia, factors associated with end-of-life practices, and quality of end-of-life care in patients with amyotrophic lateral sclerosis in the Netherlands: a population-based cohort study. The Lancet Neurology. 2023;22(7):591-601. doi:10.1016/S1474-4422(23)00155-2
New Zealand	Health New Zealand. Mortality data web tool. Accessed August 27, 2024. https://tewhatuora.shinyapps.io/mortality-web-tool/
Switzerland	Güth U, Junker C, McMillan S, Elfgen C, Schneeberger AR. Long-term experience on assisted suicide in Switzerland: dementia, mental disorders, age-related polymorbidity and the slippery slope argument. Public Health. 2023;223:249-256. doi:10.1016/j.puhe.2023.08.001
US – California, Colorado, DC, Hawaii, Maine, New Jersey, Oregon, Vermont, Washington	Centers for Disease Control and Prevention, National Center for Health Statistics. National Vital Statistics System, Mortality 1999-2020 on CDC WONDER Online Database, released in 2021. Data are from the Multiple Cause of Death Files, 1999-2020, as compiled from data provided by the 57 vital statistics jurisdictions through the Vital Statistics Cooperative Program. Accessed August 27, 2024. https://wonder.cdc.gov/ucd-icd10.html

© 2024 Heidinger B et al. *JAMA Internal Medicine*.

eTable 3. Matched narrative descriptions of the most and least common underlying diseases for mortality ICD-10 chapters and subchapters by jurisdiction

eTable 3A. Matched narrative descriptions of the most common underlying diseases for mortality ICD-10 chapters and subchapters by jurisdiction.

	**Mortality data – ICD-10 chapter or subchapter**				
**Jurisdiction**	**C00–D48 - Neoplasms**	**G00–G99 - Diseases of the nervous system**	**I00–I99 - Diseases of the circulatory system**	**J00–J99 - Diseases of the respiratory system**	**G12.2 - Motor Neurone or G12.21 - ALS**
Australia – Queensland	Cancer	Neurological disorder		Disease of the respiratory system	
Australia – South Australia	Malignancy	Neurodegenerative disease		Respiratory failure	
Australia – Tasmania	Cancer	Neurodegenerative		Respiratory	
Australia – Victoria	Cancer	Neurological		Respiratory	Motor Neurone Disease
Australia – Western Australia	Cancer related	Neurological		Respiratory-related	
Belgium	Cancer	Diseases of the nervous system	Diseases of the circulatory system	Diseases of the respiratory system	
Canada	Cancer	Neurological	Cardiovascular	Respiratory	ALS
Luxembourg	Cancer	Neurodegenerative diseases	Neurovascular diseases		
The Netherlands	Cancer	Neurological disorders	Cardiovascular disease	Pulmonary disorders	ALS
New Zealand	Cancer	Neurological condition	Cardiovascular condition	Chronic respiratory disease	
Switzerland	Cancer	Neurodegenerative diseases	Cardiovascular diseases	Lung diseases	ALS
US – California	Malignant neoplasms	Neurological disease	Cardiovascular disease	Respiratory disease	
US – Colorado	Malignant neoplasms	Progressive neurological or neurodegenerative disorders	Cardiovascular disease; Cerebrovascular disease	Chronic lower respiratory disease; Interstitial lung disease	ALS
US – DC	Cancer; Brain tumour	ALS	Congestive heart failure		ALS
US – Hawaii*					

© 2024 Heidinger B et al. *JAMA Internal Medicine*.

US – Maine	All cancers	Multiple Sclerosis, ALS, Parkinson's Disease	Cardiac	Pulmonary disease, COPD	ALS
US – New Jersey	Malignancy	Neuro-degenerative disease	Cardiovascular disease,	Pulmonary disease	
US – Oregon	Cancer (non-lung), Cancer (lung),	Neurological disease	Heart/circulatory disease	Respiratory disease [e.g., COPD],	ALS
US – Vermont	Cancer	ALS, Neurodegenerative disorders			ALS
US – Washington		Neurodegenerative	Cardiovascular	Respiratory	

Note: Included underlying diseases are quoted directly from MAID reports and translated when English reports were not available.

***Hawaii's MAID report provides the specific narrative diagnosis for each individual MAID case.

eTable 3B. Matched narrative descriptions of less common underlying diseases for mortality ICD-10 chapters and subchapters by jurisdiction.

	**E00–E90 - Endocrine, nutritional, and metabolic disease**	**F00–F99 - Mental and behavioural disorders**	**K00–K93 - Diseases of the digestive system**	**M00–M99 - Diseases of the musculoskeletal system and connective tissue**
Belgium	Diseases of the endocrine, nutritional, and metabolic system	Psychiatric conditions	Diseases of the digestive system	Diseases of the musculoskeletal system and connective tissue
The Netherlands		Psychiatric disorders		
Switzerland				Musculoskeletal diseases
US – Colorado			Chronic liver disease	
US – Oregon	Endocrine/metabolic disease [e.g, diabetes],		Gastrointestinal disease [e.g., liver disease]	

© 2024 Heidinger B et al. *JAMA Internal Medicine*.

eTable 4. MAID eligibility laws and regulations by jurisdiction

Jurisdiction	Legal method(s) of MAID*	Prognosis requirement	Age requirement
**Australia – Queensland**	PAS and Euthanasia	<6 months, <12 months for neurodegenerative condition	18
**Australia – South Australia**	PAS and Euthanasia**	<6 months, <12 months for neurodegenerative condition	18
**Australia – Tasmania**	PAS and Euthanasia	<6 months, <12 months for neurodegenerative condition	18
**Australia – Victoria**	PAS and Euthanasia**	<6 months, <12 months for neurodegenerative condition	18
**Australia – Western Australia**	PAS and Euthanasia	<6 months, <12 months for neurodegenerative condition	18
**Belgium**	PAS and Euthanasia	None	None
**Canada**	PAS and Euthanasia	None***	18
**Luxembourg**	PAS and Euthanasia	None	18
**The Netherlands**	PAS and Euthanasia	None	12
**New Zealand**	PAS and Euthanasia	<6 months	18
**Switzerland**	PAS only	None	None
**US – California**	PAS only	<6 months	18
**US – Colorado**	PAS only	<6 months	18
**US – DC**	PAS only	<6 months	18
**US – Hawaii**	PAS only	<6 months	18

© 2024 Heidinger B et al. *JAMA Internal Medicine*.

**US – Maine**	PAS only	<6 months	18
**US – New Jersey**	PAS only	<6 months	18
**US – Oregon**	PAS only	<6 months	18
**US – Vermont**	PAS only	<6 months	18
**US – Washington**	PAS only	<6 months	18

*****PAS: Physician-Assisted Suicide; Euthanasia: Physician-Administered Voluntary Euthanasia

**In Victoria and South Australia, legislation includes procedural steps making Euthanasia much more challenging to access than PAS, so that >85% of MAID recipients use PAS. This compares to Western Australia, New South Wales and Queensland, where <35% use PAS. (www.gogentleaustralia.org.au/state_of_vad_report)

***In Canada, until March 2021, MAID recipients had to be at a point where their “natural death” had become “reasonably forseeable”. There is currently no prognostic exclusion criterion in Canada, but >97% of MAID recipients still meet this criterion.

© 2024 Heidinger B et al. *JAMA Internal Medicine*.
