# Supplementary material for: International Comparison of Underlying Disease Among Recipients of Medical Assistance in Dying
Source: JAMA Intern Med. 2024 Dec 9;185(2):235–7. doi: 10.1001/jamainternmed.2024.6643 (PMC11791694; doi:10.1001/jamainternmed.2024.6643)
Supplement: Supplement 2. — Data Sharing Statement [file jamainternmed-e246643-s002.pdf]

Data Sharing Statement

Heidinger. International Comparison of Underlying Disease Among Recipients of Medical Assistance in Dying. *JAMA Intern Med*. Published December 09, 2024.
doi:10.1001/jamainternmed.2024.6643

Data
Data available: No

Additional Information
Explanation for why data not available: All data used in this study are already publicly available, as a condition of being included in this study. Therefore, the authors do not have any additional data to share other than the MAID and mortality sources already referenced within the text. We have included a list of MAID data sources in eTable 1 and referenced our mortality data sources within the text.
